# Antibiotic procurement and ABC analysis for a comprehensive primary health care clinic in the Eastern Cape province, South Africa

**DOI:** 10.4102/sajid.v35i1.134

**Published:** 2020-11-25

**Authors:** Samridhi Sharma, Roman Tandlich, Mohamed Docrat, Sunitha Srinivas

**Affiliations:** 1 Division of Pharmacy Practice, Faculty of Pharmacy, Rhodes University, Grahamstown, South Africa; 2 Division of Pharmaceutical Chemistry, Faculty of Pharmacy, Rhodes University, Grahamstown, South Africa; 3 Eastern Cape Department of Health, Grahamstown, South Africa

**Keywords:** antibiotics, antimicrobial resistance, ABC analysis, Essential Medicines List, expenditure, primary health care, procurement, South Africa

## Abstract

**Background:**

Antimicrobial resistance (AMR), a major threat to global public health, can be addressed using a managed care approach. This includes timely analysis of antibiotic consumption and procurement data to drive evidence-based policies and practices in healthcare facilities. ‘ABC analysis’ presents an opportunity for this.

**Methods:**

ABC analysis data for a comprehensive Primary Health Care (PHC) clinic in the Eastern Cape province of South Africa was obtained from the Provincial Department of Health for 01 April 2015 to 31 March 2018. Procured antibiotics were analysed on the quantities purchased, total cost, route of administration and spectrum of activity. Antibiotic categorization was also carried out according to the *World Health Organization Model List of Essential Medicines* (WHO EML) 2017.

**Results:**

Antibiotics made up approximately 7% of the total annual pharmaceutical expenditure. A total of 31, 35 and 34 antibiotics were procured in the first, second and third years, respectively. The most procured antibiotics were: (1) isoniazid, (2) flucloxacillin, (3) azithromycin, (4) a combination of rifampicin, isoniazid, pyrazinamide, and ethambutol and (5) amoxicillin. Overall, 55%, 2% and 15% of antibiotics accounted for the ‘Access’, ‘Watch’ and ‘Access and Watch’ categories, respectively, of the WHO EML. No ‘Reserve’ antibiotics were procured. The remaining 28% were antituberculosis medicines. Altogether, 89%, 8% and 3% of the antibiotics were respectively administered orally, systemically, and topically. A total of 58% were broad-spectrum and 42% were narrow-spectrum antibiotics.

**Conclusion:**

Oral antibiotics in the ‘Access’ category presented favourable usage of antibiotics. Decreasing the use of broad-spectrum antibiotics requires consideration.

## Introduction

Antimicrobial resistance (AMR) places ever-increasing burden on public health around the world, especially on countries which are classified as low- and middle-income countries (LMICs).^1,2^ If this threat is not dealt with urgently, the impacts will include a rise in annual AMR-related deaths from the current count of 700 000 to 10 million deaths annually by 2050.^3^ A majority of these deaths will be in Asia and Africa, with the actual numbers predicted as 4 730 000 and 4 150 000, respectively.^3^ In monetary terms, every person on Earth could lose around 10 000 USD in income per capita, as a result of the potential impact of AMR.^3^ The median household income could also be impacted in the negative direction.^4^ Higher-income countries, particularly those in Europe and North America, have potential resources and the required momentum to tackle AMR.^5,6^ However, because of medical, social and economic reasons, countries which are geographically located in ‘South and Southeast Asia and in Sub-Saharan Africa’ are expected to suffer more severe impacts of AMR.^5^

Several factors contribute to the level of resistance, such as the spectrum of activity and route of administration of antibiotics, which can influence the normal gut microbiota, such as *E. coli*.^7,8,9^ Even more worrying are recent reports of some *E. coli* strains having acquired resistance against antibiotics such as carbapenems.^10^ To tackle challenges resulting from acquired resistance, and to further provide a framework for the appropriate use of antibiotics and support the World Health Organization’s (WHO) *Global Action Plan on Antimicrobial Resistance*, several actions and initiatives have been underway. One of them led to international experts updating the *2017 World Health Organization Model List of Essential Medicines* (WHO EML) to include three categories of antibiotics, that is, ‘Access’, ‘Watch’ and ‘Reserve’ antibiotics. These categories have been accompanied with the recommendations for the usage and indications for antibiotics in each category. The WHO EML aims to improve treatment outcomes and decrease the development of resistance, as well as preserve the effectiveness of ‘last resort’ antibiotics.^11^

In most developing countries, pharmaceutical expenditure comes second after salaries, and can cost up to 50% of the total health expenditure.^12^ For the period 2000–2010, data indicated a 36% increase in antibiotic consumption worldwide and around 27.36% was evaluated to have originated from antibiotic consumption in Brazil, Russia, India, China and South Africa (the BRICS countries).^13^ The lack of monitoring of medicine supply systems along with shortages and stock-outs of essential medicines, including antimicrobials, lead to several negative consequences; one of them being the increased circulation of substandard or counterfeit medicines in the supply chain.^14^ Thus, managed care is extremely important, given the clinical and financial burdens of treating common infections in conjunction with the increasing emergence of AMR.^15^ The use of managed care approaches, such as ABC analysis, where the medicines purchased are categorised according to the percentage of the total medicine procurement expenditure or budget that is allocated to them, can lead to improved quality of healthcare service delivery and patient care, as well as cost-effectiveness in healthcare systems.^16,17^ ABC analysis enables informed decision-making in the pharmaceutical management cycle. This cycle consists of ‘selection, procurement, distribution and use of high-cost and high-use pharmaceuticals to optimize the use of scarce resources’.^12^

Health systems can be improved and strengthened through antimicrobial stewardship programmes and combating AMR by evaluating and monitoring antibiotic or antimicrobial consumption, that is, through better management and more appropriate antibiotic usage.^18,19^ Documenting or tracking antimicrobial consumption data is important to comprehend AMR in any particular setting, as the relevant selection pressure from antimicrobial use has been shown to be one of the main driving factors of AMR.^1^ There is a lack of consumption data being utilised for evidence-based decision making globally, including in South Africa.^3,20^ It is important to collect and report on such data as they might have links to vulnerability of the population. The healthcare systems and population in South Africa are vulnerable to AMR primarily due to the middle-income status of the country and the quadruple burden of disease.^21^ Healthcare expenditure and the maintenance of AMR stewardship programmes are linked to healthcare spending and evidence-based medicine. This is a potential problem in South Africa, where the recent negative GDP growth rate of -51% is a contributing factor in limited spending.^22,23^ This can increase the health vulnerability of the population and the South African public healthcare system, which is often under-resourced and is used by 84% of the South African population.^20^ Therefore, it is of essential importance to analyse antibiotic procurement and expenditure in these resource-limited facilities.^24^ In this study, the authors aim to analyse antibiotic procurement data and conduct ABC analysis for antibiotics at a comprehensive primary health care (PHC) clinic in Grahamstown (or Makhanda, as it is now called).

## Methods

### Setting

This study was conducted at a comprehensive PHC clinic in Grahamstown (or Makhanda) which is the largest city in the Makana Local Municipality. This local municipality is located in the Eastern Cape province of South Africa. The choice of the setting for this study was based on previous research conducted there by some of the authors of this article and also the geographical location of Rhodes University.

### Study design

A quantitative methodology was used in this study. The Eastern Cape Department of Health records pharmaceutical procurement data for the comprehensive PHC clinic. The financial year at that PHC clinic ran from 01 April to 31 March. The medicines were coded using the Internal Control Number (ICN) coding system. Upon submission of a form and the package insert, an ICN is assigned by the Department of Health to each claim for identification and tracking purposes. The procurement data were inclusive of the following standard values or parameters: ICN; Item Description; Total Issue Quantity; Total Issue Value; Percentage of Total Value; and Cumulative Percentage of Total Value. The calculations for Total Issue Value and Percentage of Total Value are shown in Equation 1 and Equation 2:

Total Issue Value=Issue Value Of Single Item×Total Issue Quality of Item [Eqn 1]

$$Percentage\;of\;Total\;Value=\left(\frac{Total\;Issue\;Value}{Total\;Pharmaceutical\;Expenditure}\right)\times 100$$ [Eqn 2]

### Antibiotic procurement analysis

Pharmaceutical procurement data were obtained from the Eastern Cape Department of Health for a period of 3 years – from 01 April 2015 to 31 March 2018 – for the comprehensive PHC clinic. All the antibiotics were selected from the list and analysed based on quantities purchased, total expenditure, route of administration and spectrum of activity.

### ABC analysis

ABC analysis is a method or a form of inventory control in which the quantity of medicines consumed and expenditures for procurement are captured and then evaluated and recorded. During the ABC analysis, the consumed medicines are categorised as belonging to either Class A or Class B or Class C. The details and relevant additional information can be seen in Table 1.^12^

**TABLE 1:** Explanation of the three different categories in ABC analysis.^12^

Description	Class of medicines in ABC analysis		
	Class A	Class B	Class C
Total expenditure (%)	75–80	15–20	5–10
Total quantity (%)	10–20	10–20	60–80
Required control	Constant control	Need-based control	Little control

*Source*: Management Sciences for Health. MDS-3: Managing access to medicines and health technologies [homepage on the Internet]. 2012 [cited 2020 Jan 16]. Available from: https://www.msh.org/sites/msh.org/files/mds3-jan2014.pdf

On the most fundamental level, ABC analysis is a method of quantitative analysis which allows one to organise the items being purchased at a facility, for example, a PHC clinic, and categorise them according to the incurred cost and procured volume. Category A consists of items that generally account for lowest procurement numbers but the highest proportion of the total expenditure. Category B falls in the middle in terms of the number of procured items and the annual expenditure of medicines at the PHC clinic. Category C consists of items that generally account for the highest procurement numbers but the lowest proportion of the total expenditure. Most procured antibiotics are also the most likely to be dispensed to patients at the PHC clinic. Tracking the expenditure on antibiotics, and the categorisation of the antibiotics according to procurement volume as a proportion of the total amount spent on medicines, can provide insights on the possible overuse or misuse of antibiotics at the PHC clinic. This type of analysis will be performed in this study, where ABC analysis is also combined with the WHO EML to investigate whether antibiotics being dispensed belong to the ‘Access’ and ‘Watch’ categories, and whether ‘Reserve’ antibiotics are used. The ABC analysis is used as a way to identify the most procured medicines – in this case antibiotics – and can help indicate target medicines for interventions in the prevention of the development of AMR.

### World Health Organization Model List of Essential Medicines

All of the antibiotics were selected from the list and analysed based on the WHO EML. Three different categories of antibiotics – ‘Access’, ‘Watch’ and ‘Reserve’ – were developed in order to support antimicrobial stewardship and reduce AMR.^11,25^ The individual categories are defined as follows: ‘Access’ antibiotics ‘treat a wide range of common infections and should be widely available, affordable and quality-assured’.^11,25^ ‘Watch’ antibiotics ‘have higher resistance potential and are only used as first- or second-choice treatments for a limited number of indications’.^11,25^ Some antibiotics in the ‘Access’ category are also included in the ‘Watch’ category, which primarily differ on the indication for use, route of administration and dosage. ‘Reserve’ antibiotics ‘are used as the “last resort” when all other alternatives have failed’.^11,25^ Application of the WHO EML and ABC analysis together can provide a more detailed picture about the procured antibiotics in the comprehensive PHC clinic as it can help optimise the antimicrobial therapy and the prevention of AMR in a given healthcare facility. The combination of these two tools can help contribute to the effective pharmaceutical management eventually leading to the rational use of antibiotics in LMICs such as South Africa.

## Results

From 01 April 2015 to 31 March 2016, a total of R2 030 550.10 (119 247.22 USD) was spent on pharmaceutical expenditure, of which R138 062.72 (8107.95 USD) was spent on antibiotics. From 01 April 2016 to 31 March 2017, a total of R2 659 997.03 (156 212.48 USD) was spent on pharmaceutical expenditure, of which R179 849.84 (10 561.96 USD) was spent on antibiotics. From 01 April 2017 to 31 March 2018, a total of R2 868 599.71 (168 463.00 USD) was spent on pharmaceutical expenditure, of which R182 322.07 (10 707.15 USD) was spent on antibiotics. Each year, approximately 7% of the total pharmaceutical expenditure was spent on antibiotics.

**FIGURE 1:**
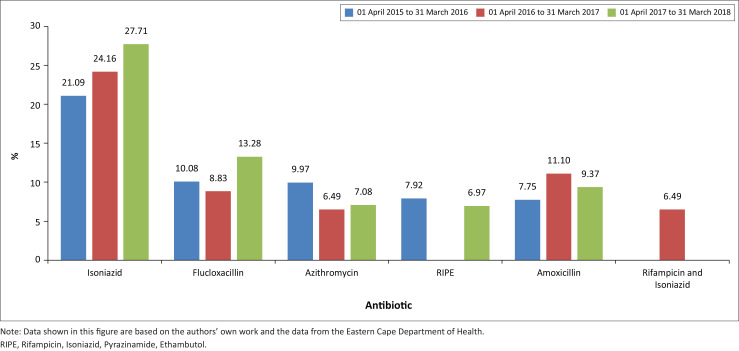
Percentage cost of total antibiotic expenditure between 01 April 2015 and 31 March 2018.

Figure 1 below compares the percentage cost of total antibiotic expenditure for the five most commonly procured antibiotics between 01 April 2015 and 31 March 2018. In the first year, isoniazid 300 mg 28’S was purchased at the largest scale and had the highest expenditure (21.09% of the total antibiotic expenditure and 1.43% of the total pharmaceutical expenditure). In the second year, isoniazid 300 mg 28’S was purchased at the second largest scale and had the highest expenditure (24.16% of the total antibiotic expenditure and 1.63% of the total pharmaceutical expenditure). In the third year, isoniazid 300 mg 28’S was purchased at the largest scale and had the highest expenditure (27.71% of the total antibiotic expenditure and 1.76% of the total pharmaceutical expenditure).

Table 2^26^ shows the indications of the five most commonly procured antibiotics. Data in Table 2 were sourced from the Standard Treatment Guidelines (STGs) and Essential Medicines List (EML) for South Africa.^25,26^

**TABLE 2:** Indications of the five most commonly procured antibiotics which are cited (verbatim) from the Standard Treatment Guidelines.^26^

Antibiotic	Indication(s)
Isoniazid	Tuberculosis
Flucloxacillin	Boil abscess, impetigo, cellulitis, acute paronychia, acute eczema, mastitis, herpes zoster (shingles), otitis externa
Azithromycin	Dental abscess, acute rheumatic fever, boil abscess, impetigo, cellulitis, acute eczema, mastitis, prostatitis, sexually transmitted infections (STIs), acute bronchitis, pneumonia in children, conjunctivitis of new born, otitis externa, acute otitis media, acute bacterial sinusitis, tonsillitis and pharyngitis, rabies, STI prophylaxis
RIPE	Tuberculosis
Amoxicillin	Dental abscess, complicated severe acute malnutrition, valvular heart disease and congenital structural heart disease, urinary tracts infections (UTIs) (e.g. complicated cystitis), diabetic foot ulcers, measles, chronic obstructive pulmonary disease, acute bronchitis, pneumonia, acute otitis media, acute bacterial sinusitis, animal and human bites (e.g. rabies)

*Source*: Department of Health Republic of South Africa. Standard treatment guidelines and Essential Medicines List for South Africa – Primary health care level. 5th ed. [homepage on the Internet]. 2014 [cited 2020 Jan 17]. Available from: http://www.kznhealth.gov.za/pharmacy/edlphc2014a.pdf

RIPE, Rifampicin, Isoniazid, Pyrazinamide, Ethambutol.

All procured antibiotics were found on the WHO EML 2017. Table 3^25^ compares the number of antibiotics per category – ‘Access’, ‘Watch’ and ‘Reserve’ – from 01 April 2015 to 31 March 2018. The remaining medicines formed part of the antituberculosis medicines in the WHO EML 2017.^25^

**TABLE 3:** Procured antibiotics are categorised according to the World Health Organization Model List of Essential Medicines.^25^

Antibiotic category	Number of antibiotics		
	01 April 2015 to 31 March 2016	01 April 2016 to 31 March 2017	01 April 2017 to 31 March 2018
Access	17	19	19
Watch	1	1	0
Access and Watch	5	5	5
Reserve	0	0	0
Antituberculosis medicines	8	10	10
**Total**	**31**	**35**	**34**

*Source:* World Health Organization. WHO Model List of Essential Medicines – 20th edition [homepage on the Internet]. March 2017 [cited 2018 Sept 23]. Available from: http://apps.who.int/iris/bitstream/handle/10665/273826/EML-20-eng.pdf?ua=1

Tables 4 and 5 compare the antibiotic route of administration and spectrum of activity from 01 April 2015 to 31 March 2018, respectively.

**TABLE 4:** Antibiotic route of administration between 01 April 2015 and 31 March 2018.

Route of administration	Number of antibiotics		
	01 April 2015 to 31 March 2016	01 April 2016 to 31 March 2017	01 April 2017 to 31 March 2018
Oral	27	31	31
Systemic	3	3	2
Topical	1	1	1
**Total**	**31**	**35**	**34**

**TABLE 5:** Antibiotic spectrum of activity between 01 April 2015 and 31 March 2018.

Spectrum of activity	Number of antibiotics		
	01 April 2015 to 31 March 2016	01 April 2016 to 31 March 2017	01 April 2017 to 31 March 2018
Broad-spectrum	18	21	19
Narrow-spectrum	13	14	15
**Total**	**31**	**35**	**34**

## Discussion

The overconsumption and irrational use of antibiotics has been reported to be a significant factor that contributes to the emergence of AMR. In spite of this finding, a major knowledge gap exists in this area as data are not systemically and consistently collected and analysed.^20,27,28^ The level of resistance across countries is partly dependent upon the different volumes and patterns of antibiotic consumption.^13^ Approximately 7% of the total pharmaceutical expenditure was spent on antibiotics at the studied PHC clinic. Isoniazid had the highest expenditure and was purchased in the largest volume. This is understandable, as South Africa has been shown to be a country ‘amongst the top 20 countries in the world with the highest estimated numbers of incident TB cases and MDR-TB cases’.^29,30^

The PHC facilities, such as the comprehensive PHC clinic in Grahamstown (or Makhanda) where this study was conducted, have formed the backbone of the South African healthcare system and have played a crucial role in the provision and delivery of essential health services to the South African population.^26^ Since 1996, personnel in the South African PHC system have had an EML and the regularly updated STGs at their disposal.^31^ The EML and STGs provide guidance for the rational use of essential medicines to achieve Universal Health Coverage.^26^ The regular use of STGs ensures quality healthcare at PHC level as well as uniformity of medical care.^32^ As part of managed care, the EML and STGs are used extensively in PHC clinics in South Africa.^33^ By adhering to the three antibiotic categories – ‘Access’, ‘Watch’ and ‘Reserve’ – from the WHO EML, healthcare professionals at PHC clinics and hospitals can better manage public health problems related to AMR’.^33,34^

All of the antibiotics and antituberculosis medicines in this study were listed in the WHO EML 2017. ‘Access’ antibiotics were predominantly used, with an increase in their use during the 3–year duration of the study. However, there was a decrease in the use of ‘Watch’ antibiotics, whilst the use of ‘Access and Watch’ antibiotics remained constant, and there was no usage of ‘Reserve’ antibiotics. There was an increase in the use of antituberculosis medicines over the years. As recommended by WHO, ‘Access’ antibiotics, including amoxicillin, were widely available to treat common infections such as pneumonia and urinary tracts infections (UTIs).^25^ Azithromycin was listed as an ‘Access and Watch’ antibiotic. It is a medicine which is often used as the first- and second-choice for the treatment of sexually transmitted infections (STIs).^25^ As a result of this model, WHO intends for the usage of ‘Watch’ and ‘Reserve’ antibiotics to be reduced to promote rational use of antibiotics. Pharmacy and Therapeutics Committees (PTCs) ensure availability and control accessibility to essential medicines, which may vary depending on the requirements of specific healthcare settings.^35^ PTCs would therefore be useful for monitoring ‘Access’, ‘Watch’ and ‘Reserve’ antibiotics so that the overuse of ‘Watch’ and ‘Reserve’ antibiotics can be identified timeously and interventions implemented to rationalise it.^26^

Strategies to combat AMR include the following approaches: limit the use of antibiotics, the use of combination therapies and antibiotic cycling, and finally only necessary and limited use of the broad-spectrum and ‘last resort’ antibiotics.^36^ In this study, oral and broad-spectrum antibiotics were predominantly used. The lack of capacity for antimicrobial sensitivity testing in rural areas results in the absence of rapid diagnostics at the point-of-care.^37,38^ In empirical antibiotic therapy, diagnosis must be based on performing bacterial cultivations where antibiotic sensitivity of a particular strain must be established. However, such tests can take about 48 to 72 hours to complete, which can lead to delay in the start of the antibiotic therapy.^37,39^ Therefore, many healthcare professionals often prefer to prescribe broad-spectrum antibiotics.^37,39^ Thus, improved rapid diagnostics at the point-of-care are important for equipping the PHC staff to be able to prevent or slow down the development of AMR through, among other things, the correct diagnosis of patients who suffer from viral infections as compared to those who suffer from bacterial infections. This in turn can facilitate and aid limiting the use of antibiotics for viral infections, reducing overuse of broad-spectrum antibiotics, and for identifying resistant strains.^40^ The Flexicult system, commonly used in Europe, consists of zoned agar plates for diagnosing UTIs and testing antibiotic susceptibility. Though Flexicult can be cultured at the point-of-care, the agar plates need to be incubated overnight before being interpreted.^41,42^ More rapid point-of-care diagnostic tests for PHC level are being developed which can be interpreted in approximately 10 minutes.^42^

There have been considerable improvements in access to antimicrobials and life expectancy, especially in LMICs.^43^ Between 1994 and 2018 in South Africa, life expectancy increased from 58 to 61 years for males and 66 to 67 years for females.^44–46^ However, the increased life expectancy could shorten again as a result of the rise of AMR. This would or can be a function of managing or balancing increased access to appropriate antibiotic or antimicrobial therapy and limiting the inappropriate use of these medicines.^43^ In 2016, South Africa reported to only have 0.818 physicians, 5.229 nursing and midwifery personnel and 0.215 dentistry personnel per 1000 population.^47^ The shortage of trained healthcare professionals further limits quality healthcare delivery, making it difficult to address AMR.^48^ Thus, the implementation of managed care by all healthcare professionals and new approaches to financing and delivering quality healthcare are required to conserve the effectiveness of existing antibiotics.^43,49^ This is more important than ever in South Africa, partly due to recent reports about the death of children in a hospital in the Gauteng province due to an outbreak of a carbapenem-resistant strain of *Klebsiella* spp.^50^

The ABC analysis, which is the focus of this study, is a critical part of pharmaceutical procurement. Pharmaceutical procurement in turn is of fundamental importance in efficient pharmaceutical management and supply.^51^ Results of the ABC analysis, as shown in this study, can provide the baseline antimicrobial procurement data analysis. Such analyses can in turn help in gaining understanding about different measurements of antibiotic consumption at the PHC level and also about the level of resistance.^20^ Regular analysis of antibiotic procurement data can be used as an influential tool in rational management of essential medicines and identifying potential medicine-use problems in order to maintain optimal usage and expenditure, in addition to managing the global health issue of AMR.^12^

Studies such as the current one by the authors are important as one of many tools to be used in the management of AMR. Such studies are also critical to manage AMR which has been gaining significance as a global health threat in the 21st century.^52^ The scope of this study will have to be expanded to investigate the antibiotic management and procurement, for example, based on the ABC analysis, in healthcare facilities of various types. This is necessary as the average population served by a single PHC facility in the district where Grahamstown (or Makhanda) is located, has been reported to range from 4275 to 6300 in the 2003–2004 financial year.^53^ This number was estimated to have changed to around 13 000 per PHC facility in Makana Local Municipality, which Grahamstown (or Makhanda) is part of, by the 2017–2018 financial year.^54^ Documents such as staffing guidelines published by the South African Medical Research Council^55^ provide important recommendations which can be used for such research.

There is one limitation of the study which needs to be discussed before the conclusion of this article. Antibiotics that were previously classified under the ‘Access and Watch’ category by WHO have now been placed under the ‘Access’ or ‘Watch’ categories in the WHO EML 2019. This action may have been taken to better manage and monitor the use of antibiotics as well as to further reduce the use of ‘Watch’ and ‘Reserve’ antibiotics. Data for procurement here has been evaluated according to the WHO EML which was published in 2017 by WHO. In the authors’ opinion, this is justified, as the ABC analysis was performed for the 2015–2018 time period. In addition, the data from 2019 until present will have to be examined and analysed because of recent changes made to the classification of ‘Access’, ‘Watch’ and ‘Reserve’ antibiotic categories in the WHO EML 2019.^56^ The factors that led to this change are outside of the time frame of the data analysed in this study and will thus be incorporated into future and ongoing ABC analyses of antibiotic procurement in the province of the study, that is, the Eastern Cape, South Africa.

The antibiotics procured at the PHC clinic, which were classified under the ‘Access and Watch’ category in this study, have now been placed under the ‘Watch’ category in the WHO EML 2019. These antibiotics include azithromycin, cefixime, ceftriaxone and ciprofloxacin. Flucloxacillin, which was part of the ‘Access’ category, has now been removed from the WHO EML 2019.^56^ These changes have resulted in the overuse of ‘Access and Watch’ antibiotics, which could further increase the development of AMR at the PHC level. Adherence to the WHO EML 2019 would lead to reduced usage of ‘Access and Watch’ antibiotics due to their high chance of resistance.

## Conclusion

Each year at the comprehensive PHC clinic, approximately 7% of the total pharmaceutical expenditure was being spent on antibiotics, of which isoniazid had the highest expenditure. Orally administered and broad-spectrum antibiotics being predominantly utilised. It is therefore important to decrease the use of broad-spectrum antibiotics in order to tackle AMR. All procured antibiotics were found on the WHO EML 2017. As recommended by WHO, most of the procured antibiotics were part of the ‘Access’ antibiotics group, whilst none of the ‘Reserve’ antibiotics were procured. Findings from this study, the combination of the ABC analysis and the WHO classification, along with additional methodologies, can be used to monitor antibiotic procurement and so contribute to effective management of AMR.
